# MRI of Neuronal Recovery after Low-Dose Methamphetamine Treatment of Traumatic Brain Injury in Rats

**DOI:** 10.1371/journal.pone.0061241

**Published:** 2013-04-18

**Authors:** Guang Liang Ding, Michael Chopp, David J. Poulsen, Lian Li, Changsheng Qu, Qingjiang Li, Siamak P. Nejad-Davarani, John S. Budaj, Hongtao Wu, Asim Mahmood, Quan Jiang

**Affiliations:** 1 Department of Neurology, Henry Ford Hospital, Detroit, Michigan, United States of America; 2 Department of Biomedical and Pharmaceutical Sciences, University of Montana, Missoula, Montana, United States of America; 3 Department of Neurosurgery, Henry Ford Hospital, Detroit, Michigan, United States of America; 4 Department of Physics, Oakland University, Rochester, Michigan, United States of America; Wayne State University, United States of America

## Abstract

We assessed the effects of low dose methamphetamine treatment of traumatic brain injury (TBI) in rats by employing MRI, immunohistology, and neurological functional tests. Young male Wistar rats were subjected to TBI using the controlled cortical impact model. The treated rats (n = 10) received an intravenous (iv) bolus dose of 0.42 mg/kg of methamphetamine at eight hours after the TBI followed by continuous iv infusion for 24 hrs. The control rats (n = 10) received the same volume of saline using the same protocol. MRI scans, including T2-weighted imaging (T2WI) and diffusion tensor imaging (DTI), were performed one day prior to TBI, and at 1 and 3 days post TBI, and then weekly for 6 weeks. The lesion volumes of TBI damaged cerebral tissue were demarcated by elevated values in T_2_ maps and were histologically identified by hematoxylin and eosin (H&E) staining. The fractional anisotropy (FA) values within regions-of-interest (ROI) were measured in FA maps deduced from DTI, and were directly compared with Bielschowsky’s silver and Luxol fast blue (BLFB) immunohistological staining. No therapeutic effect on lesion volumes was detected during 6 weeks after TBI. However, treatment significantly increased FA values in the recovery ROI compared with the control group at 5 and 6 weeks after TBI. Myelinated axons histologically measured using BLFB were significantly increased (p<0.001) in the treated group (25.84±1.41%) compared with the control group (17.05±2.95%). Significant correlations were detected between FA and BLFB measures in the recovery ROI (R = 0.54, p<0.02). Methamphetamine treatment significantly reduced modified neurological severity scores from 2 to 6 weeks (p<0.05) and foot-fault errors from 3 days to 6 weeks (p<0.05) after TBI. Thus, the FA data suggest that methamphetamine treatment improves white matter reorganization from 5 to 6 weeks after TBI in rats compared with saline treatment, which may contribute to the observed functional recovery.

## Introduction

Traumatic brain injury (TBI) is a leading factor of morbidity and mortality in Western countries [1], [2], and an important public health problem. There is an urgent need to develop a novel approach for the treatment of TBI, that can restore brain function. TBI produces a primary insult, which may trigger widespread brain damage regardless of the original site of injury [3]. Thus, patients with TBI may suffer from axonal degeneration and generalized cerebral atrophy associated with poor neurological functions [4]–[8]. Unfortunately, the management of patients with TBI to-date has primarily focused on therapeutic intervention designed to reduce cellular damage and brain edema [9]; currently, no effective clinical treatment can repair biostructural damage to the neurons and prevent or reduce secondary pathological processes [10]. However, experimental pharmacological and cell-based treatments of TBI which promote brain remodeling have shown promising results in improving functional recovery after TBI in animals [11]–[15].

Methamphetamine is a potent psychostimulant that alters the release and reuptake of dopamine, norepinephrine, and serotonin. Large doses of methamphetamine result in neuronal toxicity and the induction of cell death throughout the brain [16]. However, low dose methamphetamine may be protective after oxygen glucose deprivation by decreasing excitatory synaptic potential, and prevents cell death by modulating cytokines and neurotrophic factors [17], [18]. Recently, in a lateral fluid percussion injury model of rat, low dose methamphetamine treatment at three hours after TBI significantly increased the presence of immature neurons, and reduced apoptotic cell death, as well as improved both behavioral and cognitive dysfunctions [15].

Prior methamphetamine studies focused on histological investigation [15], [17], [18]. The therapeutic effects of low dose methamphetamine treatment of TBI can be dynamically mornitored and longitudinally investigated using magnetic resonance imaging (MRI) [19], [20]. MRI, including T2-weighted imaging (T2WI), susceptibility weighted imaging (SWI), diffusion tensor imaging (DTI) and cerebral blood flow (CBF) measurement with the arterial spin labeling (ASL) technique, provides a preferable way to track biostructural damage and changes of the brain after TBI [19], [20]. DTI permits assessment of diffuse axonal injury after brain injury [21]–[23]. Fractional anisotropy (FA) deduced from DTI is reduced in major white matter tracts in the central areas of the injured brain [21], [22], and is increased in the recovery regions surrounding the impact core after cell-based treatment of TBI in rats. This increase in FA has been attributed to axonal remodeling and increased oligodendrocyte generation [19]. Thus in the present study, we employed MRI to assess the effects of low dose methamphetamine treatment on neuronal recovery following a controlled cortical impact (CCI) model of TBI in rats.

## Materials and Methods

All experimental procedures were conducted and performed in accordance with guidelines for animal research under a protocol approved by the Institutional Animal Care and Use Committee of Henry Ford Hospital (No.1051). MRI scan and data analysis, TBI surgery and methamphetamine treatment, neurobehavioral functional tests and histological measurements, were performed in a double-blind fashion. Three rats died within the 6 week test period and before completion of all MRI scans and were excluded from measurement and analysis.

### TBI Model

Young male Wistar rats (Charles River, Wilmington, MA) weighing 200–300 g at arrival were kept at the animal facility for 7 days before initiating the experiments. Rats were anesthetized with chloral hydrate (350 mg/kg). Body temperature was maintained at 37°C with a feedback-regulated water recirculating pad. Blood gases and blood pressure were monitored during the surgical procedure by placing PE-50 catheters into the femoral artery and vein to obtain blood. The head of each rat was then mounted in a stereotaxic frame in a prone position and secured by ear bars and an incisor bar. Two 10 mm-diameter craniotomies were performed adjacent to the central suture, midway between the lambda and the bregma, leaving the dura mater over the cortex intact. The left craniotomy confined the location of experimental impact while the right one allowed for the lateral movement of cortical tissue. Injury was induced by a pneumatic impact device [24] on the intact dura. A single strike was delivered at 4 m/sec with a 2.5 mm of compression to the left cortex with a pneumatic piston containing a 6 mm-diameter tip. After the impact, the bone plate was not replaced and was sealed with bone wax, the skin was then sutured with 4-0 surgical thread. Buprenex (0.05 mg/kg) was subcutaneously administered to animals after brain injury for analgesia. This animal model is a clinically relevant model of TBI, which produces a severe TBI with contusion lesion affecting part of the motor and primary somatosensory cortex, and has been used for many years [25]. Animals were provided with 10 mL warmed saline via subcutaneous injection immediately after surgery and monitored 3–4 times daily within the first 48 hours following the insult and had free access to food and water.

### Methamphetamine Treatment

Eight hours after the TBI, rats were anesthetized by isofluorane inhalation. An incision was made at the inguinal crease to expose the femoral vein. An Alzet osmotic pump (DURECT Corp, Cupertino, CA) connected to a catheter was placed within the crease with the catheter extending from the pump and placed into the femoral vein. The incision was closed with 4-0 vicryl sutures. One group of rats, referred to as the treated group (n = 10), were treated with a bolus dose of 0.42 mg/kg methamphetamine via the saphenous vein followed by continuous infusion intravenously (iv) with 0.05 mg/kg/hr for 24 hrs. Animals, assigned as the control group (n = 10), received the same volume of saline as bolus iv dose followed by continuous iv infusion with 6.6µL/hr for 24 hrs. At 48 hours after pump placement, rats were again briefly anesthetized with isoflurane, the pump and femoral vein catheter were removed, and the incision in the inguinal area was sutured with 4-0 absorbable suture and sealed with skin glue.

### MRI Measurements

MRI measurements were performed with ClinScan 7T system, which combines Bruker-Biospin hardware (Bruker-Biospin, Ettlingen, Germany) interfaced to Siemens software, Syngo (Siemens, Erlanger, Germany). A birdcage type coil was used as transmitter and a quardrature half-volume coil as receiver. MRI scans were performed one day before TBI, 1 and 3 days post TBI and then weekly for 6 weeks. Pulse sequences included T2WI, SWI, and DTI. A fast gradient echo imaging sequence was used for reproducible positioning of the animal in the magnet at each MRI session. Stereotactic ear bars were used to minimize movement during the imaging procedure. During MRI measurements, anesthesia were maintained using medical air (1.0 L/min) and isoflurane (1.0–1.5%). Rectal temperature was maintained at 37°C using a feedback controlled water bath.

T2WI was acquired using a Carr-Purcell-Meiboom-Gill multislice and multiecho (six echoes) sequence. A series of six sets of images (13 slices for each set) were obtained using echo times (TEs) of 15, 30, 45, 60, 75, and 90 ms and a repetition time (TR) of 4.5 sec. Images were produced using a 32×32 mm^2^ field-of-view (FOV), 1 mm slice thickness, 128×128 image matrix. T_2_ map was obtained by exponentially fitting the six images pixel-by-pixel with different echo times for each slice. SWI employed a specialized 3-dimensional gradient echo sequence with TE = 10 ms, TR = 40 ms, flip angle of 15°, 32×32×24 mm^3^ FOV, 256×192×64 matrix (a resolution of 125µm×167µm×375µm), and flow compensation in all three directions. DTI was acquired using a spin-echo sequence with pulsed diffusion weighted gradients and echo-planar readout. The field of view was 32×32 mm^2^; 128×128 imaging matrix, 1 mm slice thickness with 13 slices, TR = 10 s and TE = 50 ms, 1 shot, 2 averages, 1 DTI baseline of b = 0 s/mm^2^, 20 directions of diffusion gradients with b = 900 and 1800 s/mm^2^, respectively, for each slice.

### Ex vivo MRI Measurements

Q-ball based DTI [26] was performed using a pulsed gradient spin echo sequence with the following parameters: 128×128 matrix; 32×32 mm^2^ FOV; 1 mm slice thickness with 13 slices; TR = 1.5 s; TE = 38 ms; δ = 10 ms; Δ = 18 ms; b = 900 s/mm^2^ at 128 directions and one baseline with b = 0; four signal average; total acquisition time was approximate 27 hours for Q-ball imaging.

### Neurobehavioral Functional Tests

Neurological function was monitored by modified neurological severity scores (mNSS) [27] and foot-fault test [28]. Baseline neurological function was established for all animals prior to TBI and again on days 1, 7, 14, 21, 28, 35, and 42 post brain injury. On day 38 to day 42 following TBI, the Morris Water Maze (MWM) test was performed to assess the impact of methamphetamine on cognitive function (learning and memory) [29].

### Immunohistochemical Staining

Animals were euthanized after completing the last MRI measurements and functional tests at 42 days post TBI. Rats were anesthetized with intraperitoneal injection with chloral hydrate, and perfused transcardially with saline, followed by 4% paraformaldehyde. Brains were isolated, post-fixed in 4% paraformaldehyde for 2 days at room temperature, and then processed for paraffin sectioning. Using light microscopy and a laser scanning confocal microscope (Zeiss LSM 510), we measured a composite index of independent measurements from multiple immunohistochemical stainings for axons (Bielschowsky’s silver and Luxol fast blue, BLFB [30]; monoclonal antibody to non-phosphorylated neurofilaments, SMI-32 [31]), dendrites (microtubule-associated protein 2, MAP-2 [32]) and synapses (synaptophysin, SYP [33]). Data were expressed as density, or percentage of area of immunoreactive proteins, to the area of the FOV. Lesion areas were measured as a percentage to the ipsilateral hemisphere areas with hematoxylin and eosin, H&E [34], immunohistochemical stained slices.

### MRI Data and Statistical Analysis

MRI image analysis was generally performed with homemade software, Eigentool [35]. Q-ball DTI analysis was performed using Camino software [36].

The TBI lesion was identified on the MRI T_2_ map. The ipsilateral lesion area on each slice of the T_2_ map was specified by those pixels with a T_2_ value higher than the mean plus the twice standard deviations provided by the normal tissue of the same animal on the pre-TBI T_2_ map. Lesion volume was obtained by adding all the areas measured on individual slices and multiplying by the slice thickness. The volume of the lateral ventricle was measured on T_2_ maps at a fixed structural location presented by four contiguous coronal T_2_ slices using the same criterion as described above to identify the ventricular area on each slice. The ventricular volume was obtained by adding all the areas measured on individual slices and multiplying by the slice thickness.

Two MRI regions of interest (ROI) were identified as the TBI core and recovery areas for analysis of MRI parameters. The first ROI, referred to as, the TBI core, was demarcated on the T_2_ map obtained 6 weeks after TBI, by using the T_2_ value threshold of mean plus two standard deviations based on the T_2_ value measured in the pre-TBI T_2_ map for each animal [19]. The second ROI, referred as the, TBI recovery area [19], was demarcated by subtracting the TBI core from the TBI lesion area in T_2_ maps obtained 24 hrs after TBI.

SWI was employed to identify hemorrhagic lesions after TBI and to demarcate the TBI lesion from the ventricle in rat brain [20].

The FA map from DTI was first warped to the corresponding T_2_ map; then, the TBI core and recovery ROIs obtained from the same slice of T_2_ map were, respectively, loaded onto the FA map for measurement. The present FA data were normalized to pre-TBI FA values.

Diffusion standard deviation (SD) map [37], derived from Q-ball imaging, was created based on calculating the deviation of diffusivity from a sphere for each voxel in the image. If diffusion is constrained by tubular structures, the SD will possess a non-zero value based on the complexity of the structure [37].

A mixed model, analysis of variance (ANOVA) and covariance (ANCOVA) was performed. The analysis started testing the treatment group and time (without baseline time point) interaction, followed by testing the group difference at each time point, or the time effect for each treatment group, if the interaction or the overall group/time effect was detected at the 0.05 level. Subgroup analysis was followed to test the difference at each group and time level using the paired two-tailed *t*-test. Two-sample *t*-test was performed to check the balance of the data at the baseline, as well as to test the treatment effect at day 42 post TBI for histological evaluation. Pearson’s correlation analysis was applied between MRI values obtained at 6 weeks after TBI and histological measurements. MRI and histological measurements are summarized as mean and standard error (SE).

## Results

**Figure 1:**
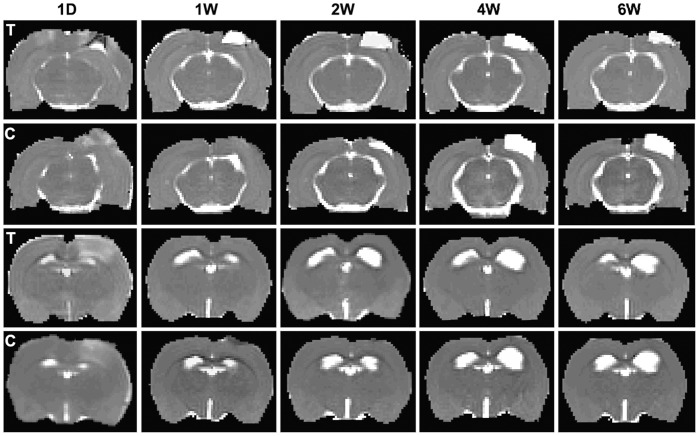
T_2_ maps demonstrating the evolution of TBI lesion volumes (two upper rows) from representative low dose methamphetamine treated (T) and saline treated (C) TBI rats. The two lower rows are also T_2_ maps, which demonstrate the evolution of ventricular expansion for treated (T) and control (C) rats. Although the T_2_ maps obtained at 6 weeks after TBI showed smaller lesion and ventricular volumes in the treated rats (the 1^st^ and 3^rd^ rows) compared with the control rats (the 2^nd^ and 4^th^ rows), no statistically significant differences were found between the treated (*n = *10) and control (*n = *10) groups during the 6 weeks after TBI.

**Figure 2:**
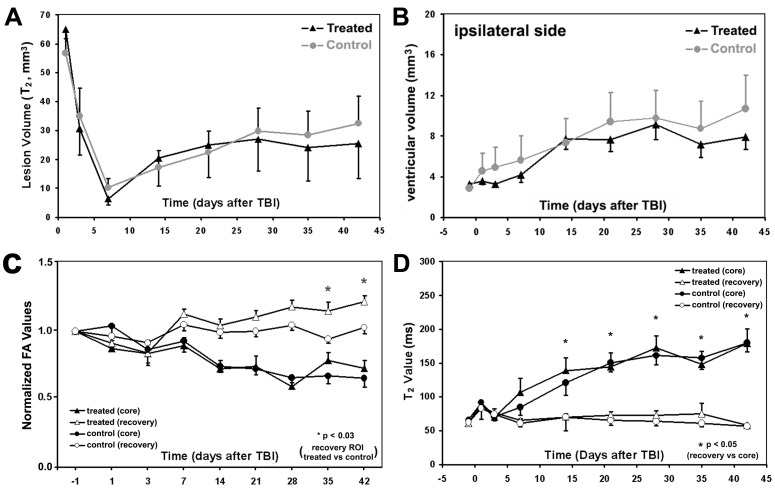
Lesion (A) and ipsilateral ventricular (B) volumes measured on T_2_ maps after TBI were charted for both treated (*n = *10) and control (*n = *10) rats. Although the lesion and ventricular volumes were smaller in the treated rats, i.e. evident at 4 weeks for lesion and 3 weeks for ventricle after TBI, than that in the control rats, no statistically significant differences were found between the two groups during the 6 weeks after TBI. Normalized FA values (C) of recovery area demonstrated significant differences between the groups starting 3 weeks after TBI in rats. T_2_ values of both core and recovery regions (D) did not show statistical differences between the two groups in 6 weeks after TBI; however, significant differences were observed between core and recovery regions starting 2 weeks after TBI for all rats.

The lesion volumes of TBI damaged cerebral tissue were demarcated by elevated values in T_2_ maps and measured at 24 hrs, 72 hrs and weekly from 1 to 6 weeks post TBI in rats. T_2_ maps of representative rats are shown in figure 1, which depict the evolution of T_2_ values for a methamphetamine treated rat (the 1^st^ row) and a saline treated rat (the 2^nd^ row), respectively. Lesion volumes at 24 hrs after TBI, 65.03±8.54 mm^3^ for the treated rats and 56.71±5.15 mm^3^ for the control rats, exhibited no initial statistical difference (p>0.41) between the two groups. At 6 weeks after TBI, lesion volumes were 25.51±12.14 mm^3^ for the treated, and 32.49±9.45 mm^3^ for the control, respectively, and no statistical differences (p>0.65) were detected. Methamphetamine treatment did not significantly reduce lesion volumes compared to the controls during 6 weeks after TBI in rats, and the lesion volumes are quantitated in Fig. 2 a.

The two lower rows of T_2_ maps in figure 1, from a methamphetamine treated rat (the 3^rd^ row) and a saline treated rat (the 4^th^ row), respectively, exhibited the expansive profiles of cerebral ventricles after TBI. Pre-TBI, the lateral ventricular volumes were 3.12±0.09 mm^3^ in each hemisphere and were demarcated using T_2_ maps. All animals exhibited similar ventricular volumes on both hemispheres, and no differences were detected between the two treatment groups. After TBI, ventricular volumes were measured at 24 hrs, 72 hrs, and then weekly from 1 to 6 weeks. No benefits of low dose methamphetamine treatment were observed on reducing ventricular expansion in the treated rats, compared to the controls (Fig. 2 b). At 6 weeks after TBI, the ipsilateral ventricular volumes were measured and found to be 7.93±1.29 mm^3^ for the treated rats, and 10.71±3.32 mm^3^ for the control animals. Correspondingly, in the contralateral side, the ventricular values were 4.55±1.00 mm^3^ for the treated and 5.29±1.20 mm^3^ for the control groups. No significant differences were detected between the two groups (p>0.45 for ipsilateral side, p>0.64 for the other side).

The temporal profiles of FA values, normalized to pre-TBI measurements, of the TBI core and recovery ROIs were calculated (Fig. 2 c) and used for analysis. The FA values obtained from the TBI core ROIs remained low within six weeks after TBI for both control and treated groups, and no significant differences were detected in FA measurements of the core regions between the two groups. However, the FA values in the TBI recovery ROIs increased during 6 weeks post TBI in both control and treated groups. The methamphetamine treated group revealed larger increases than the control group. Significant increases of FA values obtained from the TBI recovery ROIs were detected between the treated and control groups starting at 5 weeks after TBI. The values of the normalized FA for treated and control groups were 1.13±0.21 versus 0.94±.09 at 5 w (p<0.03), and 1.21±0.14 versus 1.03±0.15 at 6 w (p<0.02), respectively, after TBI.

T_2_ values of cerebral tissue were measured in the TBI core and recovery ROIs for all animals in the study (Fig. 2 d). There were no significant differences between the methamphetamine and saline treated animal groups for either TBI core or recovery regions, respectively. The temporal changes of T_2_ values of injured cerebral tissue, however, demonstrated a significant time effect after TBI for both groups. Starting at 14 days post TBI, T_2_ values of cerebral tissue were significantly different between the TBI core and recovery regions for both animal groups throughout the 6 week measurement time (p<0.05).

**Figure 3:**
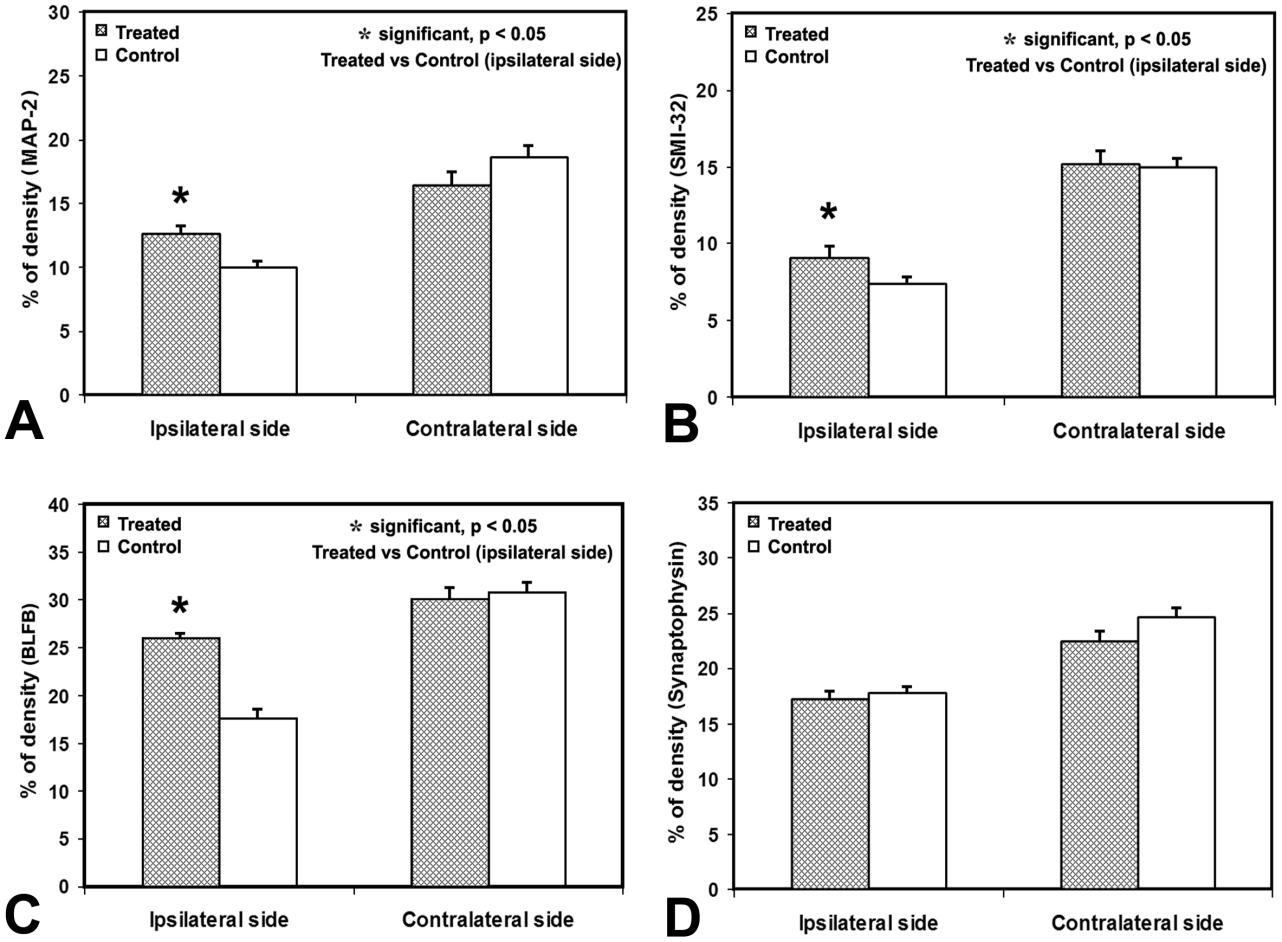
Measurements of densities of MAP-2 staining (A), SMI-32 staining (B), and BLFB staining (C), demonstrated that low dose methamphetamine treatment of TBI significantly increases neurons density and axonal reorganization in the ipsilateral hemisphere in rats. However, no differences were found for synaptophysin staining (D). No differences were found with immunohistochemical density stainings in the contralateral hemisphere of rat brain after TBI.

For the immunohistochemical measurements, significant increases in MAP-2 (Fig. 3 a, p<0.02) and SMI-32 (Fig. 3 b, p<0.02) were observed in the ipsilateral TBI lesion boundary in the treated rats with the values of 12.58±2.12% (MAP-2) and 9.09±2.25% (SMI-32), compared to the control rats with the values of 9.98±1.68% (MAP-2) and 7.36±1.52% (SMI-32), respectively. Moreover, BLFB stained axons and myelin, respectively, (Fig. 3 c) were histologically measured in the TBI boundary ROI for changes in cerebral white matter. A significant increase (p<0.001) was detected in the treated group, 25.94±1.41%, compared with control group, 17.05±2.95%. However, no significant differences were detected in synaptophysin expression between the treated, 17.27±5.44%, and the control rats, 17.82±5.44% (Fig. 3 d). Analysis of H&E sections, showed percent lesion volumes of 10.4±4.8% for the treated rats and 14.3±5.2% for the control, and no differences were detected between the two groups (p = 0.10).

A significant Pearson’s correlation was detected between normalized FA values obtained at 6 weeks after TBI and measurements of BLBF staining in TBI boundary ROI (R = 0.54, p<0.02).

**Figure 4:**
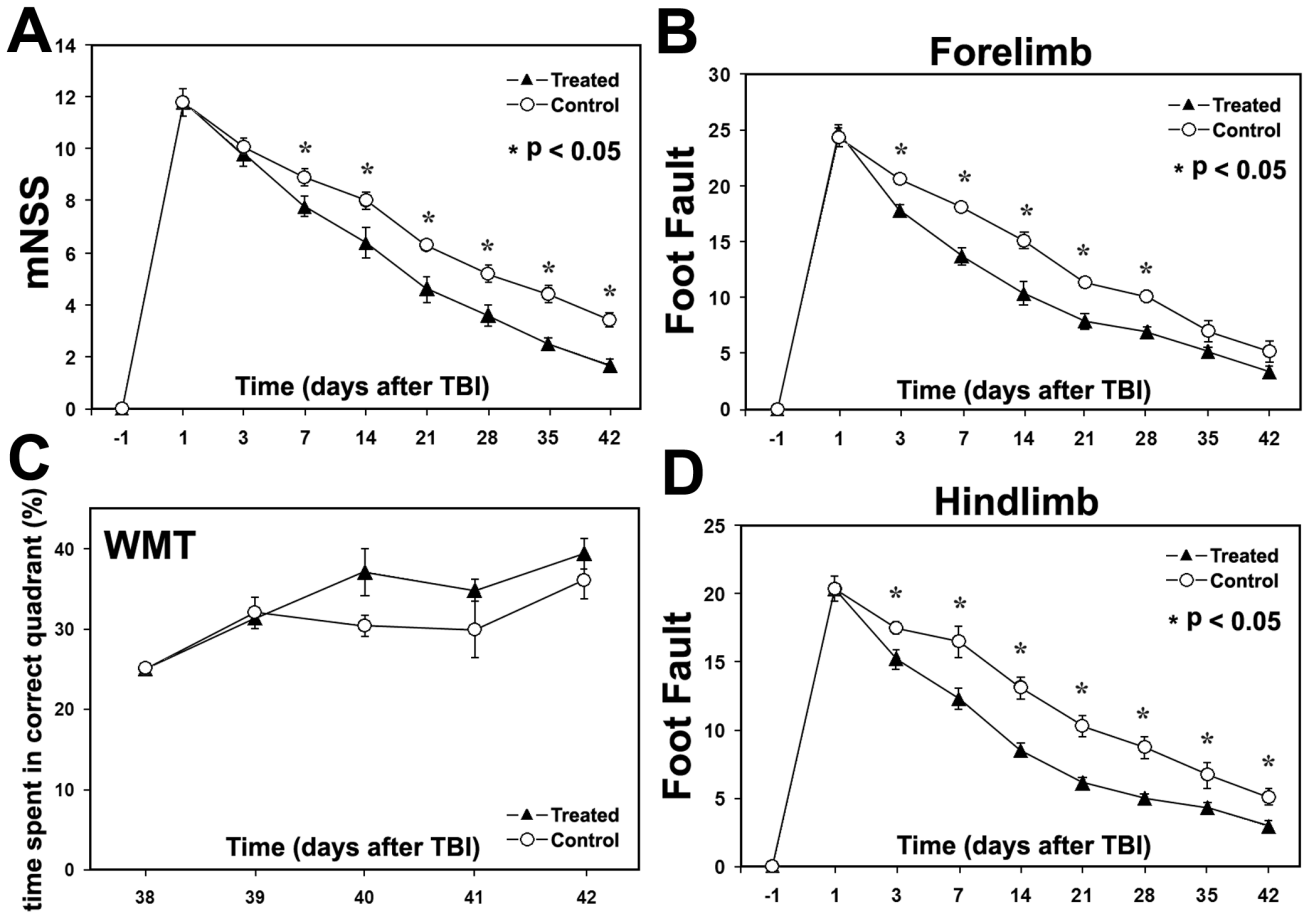
Functional tests demonstrated that low dose methamphetamine treatment significantly lowered mNSS (A) and reduced foot faults (C: forelimb; D: hindlimb) in treated rats starting at 1 week after TBI. However, no differences were observed in Morris water maze test (B) between the treated and control rats.

Reduced functional impairments were detected in the low dose methamphetamine treated group as compared with the control group. Significant improvements by methamphetamine treatment were detected in mNSS from 2 to 6 weeks (p<0.05), as shown in Fig. 4 a; and in foot-fault tests from 3 days to 6 weeks after TBI (p<0.05), as shown in Fig. 4 c for the hindlimb and Fig. 4 d for the forelimb. However, no significant improvement was detected with Morris Water Maze test, see Fig. 4 b (p = 0.12).

**Figure 5:**
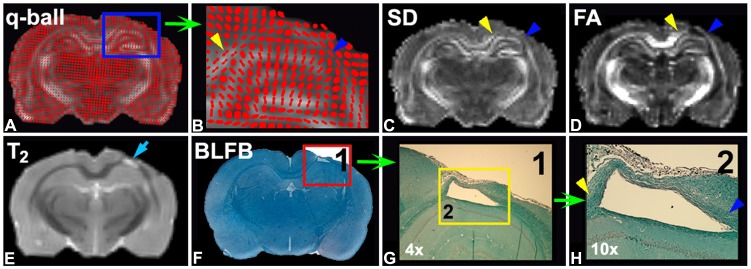
An ex vivo MRI scan of a control rat q-ball map (A), which is enlarged (B) to view fiber crossing, showed few crossing fibers (blue and yellow arrow heads) surrounding the TBI lesion core (E, cyan arrow). Both SD (C) and FA (D) maps detected the reorganized white matter (blue and yellow arrow heads), which was consistent with BLFB staining (blue and yellow arrow heads in H; G: 4× enlarged, red frame in F; H: 10× enlarged, yellow frame in G).

Figure 5 shows ex-vivo MRI from a representative control rat sacrificed at 6 weeks after TBI. The fiber orientation map, Fig. 5 a, was derived from a Q-ball MRI scan. The area within the blue frame is enlarged (Fig. 5 b), and indicates well-oriented fibers (few crossing patterns) in cortex (blue and yellow arrow heads) surrounding the TBI lesion core, that is referred to as the T_2_ map (cyan arrow in Fig. 5 e). With elevated values, both SD (Fig. 5 c) and FA (Fig. 5 d) maps identified the white matter fiber tracts along two sides of the lesion core (blue and yellow arrow heads in Fig. 5 c & d), and the FA map exhibited a higher image contrast than the SD map. The areas with reduced differences between FA and SD maps may reflect the reorganization of axonal fibers taking place after TBI, and the well organized fibers were oriented in a single direction. These MRI outcomes were consistent with the histological results obtained with BLFB staining (blue and yellow arrow heads in Fig. 5 h, enlarged from Fig5. g & f).

## Discussion and Conclusions

This study demonstrated that low dose methamphetamine treatment starting at 8 hours after traumatic insult in rats significantly improved the recovery of behavioral functions in rats by lowering mNSS and foot-fault scores after TBI, compared with saline treatment. The treatment promoted neuronal recovery after TBI, which was detected by MRI measurement of FA and confirmed by immunohistochemical outcomes. The mean relative FA values of the TBI recovery area exhibited significant increases in the treated group at 5 and 6 weeks after TBI, compared with the control group (p<0.03). The increased FA, indicating a more reorganized white matter, may contribute to the functional recovery after TBI in rats.

FA values have large variations dependent on the location of the ROI in rat brain. Using the normalized FA, referred to as, pre-TBI FA values, of the same ROI and the same animal, may provide a more precise measure of white matter changes after TBI in rat with or without treatments, since each ROI location differed among the animals.

Based on the evolution of T_2_ values measured from all animals in the study, it was reasonable to demarcate TBI damaged cerebral tissue into core and recovery tissue by using T_2_ maps obtained at 24 hours and 6 weeks after TBI. Fig. 2 d indicates that T_2_ values of cerebral tissue in core ROI, independent of treatment after TBI, increased from 1 week after TBI and then retained elevated values, while T_2_ values of cerebral tissue in recovery ROI decreased during the first week after TBI and remained the same for 6 weeks. T_2_ values were very similar and no differences were observed between the treated and control groups for either core or recovery ROIs, respectively. Meanwhile, T_2_ values of cerebral tissue exhibited significant differences (p<0.05) between the core and recovery ROIs starting at 2 weeks after TBI for both groups. Furthermore, T_2_ values of cerebral tissue from the core ROI approached the T_2_ values of cerebral spinal fluid, and T_2_ values from recovery ROI tended towards pre-TBI values. These results indicated that cerebral tissue in the core ROI may be irreversibly damaged after TBI, independent of methamphetamine or saline treatments, which suggest that effective treatment of TBI may be attributed to improved biophysical functions of cerebral tissue in the recovery region.

MRI measurements of T_2_ maps did not detect the therapeutic effects of the low dose methamphetamine treatment on reducing lesion volume (core size) and ventricular expansion up to 6 weeks after TBI in rat, although the mean values of lesion volume and expansion rate of ventricular volume were decreased (p = 0.20 for lesion volume and p = 0.32 for ventricular volume) in the treated group as compared to the control group (Fig. 2 a & b). This result was consistent with histological H&E measurements of TBI lesion volumes. The neuroprotective effect of the low dose methamphetamine treatment of stroke is dose and time dependent [18]. A prior study indicates that immediate treament of embolic stroke in rats with methamphetamine for 24 hours at a dose of 0.1 mg/kg/hr, or treatment initiated at 12 hours with a dose of 1.0 mg/kg/hr did not reduce infarct volume [18]. Thus, the curent results in TBI are consistent with the previous results, that no statistical neuroprotective effects were observed in the delayed methamphetamine treatment in stroke. Expansion of the ipsilateral ventricular volume after brain injury may indicate the loss of cerebral tissue, and ventricular volume is a sensitive MRI index of brain atrophy after stroke in rats [38], [39]. In previous experimental studies of embolic stroke, therapeutic effects of delayed Erythropoietin treatment in adult rats or Sildenafil treatment in aged rats were detected starting at 4 weeks or 6 weeks after stroke by the reduced expansion rate of the ipsilateral ventricular volume demarcated using MRI T_2_ maps, respectively [38], [39]. For TBI rats, experimental studies demonstrated a significant increase of lateral ventricular volume at one year [40], and a significantly reduced increase of lateral ventricular volume starting at 3 weeks post TBI in bone marrow stromal cell treated rats, compared to saline treated rats [20]. The results of the present study, thus, suggest that methamphetamine treatment, initiated at 8 hours after TBI with the selected dose (0.05 mg/kg/hr), does not reduce brain atrophy within 6 weeks post traumatic insult.

The methamphetamine treatment of TBI rats in the present study does not have significant effects on neuroprotection, but enhances neurorestoration (neuronal and functional improvement), similar to the delayed pharmacological treatments of stroke in rats [38], [39]. As in stroke studies [23], [37]–[39], the delayed treatments of TBI promoted neurorestorative process in cortical tissue, such as axonal density and reorganization, which fosters improved behavioral function. Without reducing lesion volume, cell-based treatments of stroke in rat also improved functional recovery from the short term (4 weeks) to the long term (1 year) [41], [42]. Therefore, the delayed low dose methamphetamine treatment of TBI in rats may improve functional outcome by enhancing biophysical functions of cerebral tissue in the recovery region, although the treatment did not reduce lesion volume and brain atrophy within 6 weeks post TBI.

Extensive axonal degeneration is the common result of traumatic brain injury [4], [6]. Injury, however, inversely induces axonal sprouting [43], [44], and new synaptic connections and myelination are present in both injured and neighboring regions [45]. White matter is reorganized, especially in the areas of the corpus callosum. Proper treatment of TBI may enhance brain reparative processes [19], [20], similar to stroke [23], [38], [39]. MRI FA was able to monitor well-reorganized white matter recovery, and similar results have been described for the detection of white matter recovery after treatment of stroke in rats [19], [23]. In the present study, our data demonstrated a remarkable increase in FA of the white matter extending from the corpus callosum to the boundary of the TBI lesion in the treated animals, in contrast to the controls (Fig. 2 c), suggesting that low dose methamphetamine treatment facilitates white matter reorganization involving the corpus callosum.

FA may be unable to detect white matter reorganization when the white matter fiber bundles cross, and FA merely expresses an overall lower value because of the assumption of a Gaussian diffusion inherent to the tensor model [46]–[48]. Solving the orientation distribution function (ODF), which is used to describe the directionality of multimodal diffusion in regions with complex fiber architecture, permits more accurate detection of crossing fibers [26], [36], [49]. This calculation involves a complex set of Q-space DTI analysis, including diffusion spectrum imaging (DSI) [49], Q-ball [26] and persistent angular structure MRI (PASMRI) [36]. In addition, diffusional kurtosis imaging (DKI) has been introduced as a quantitative measure of the degree to which the diffusion displacement probability distribution deviates from a Gaussian form [50]. Indeed, DKI has been shown to provide the information necessary to solve the ODF [51], whereas the apparent kurtosis coefficient (AKC) can provide additional information on neural tissue micro-architecture [52], such as cellular compartments and membranes, and AKC is more sensitive to axonal density than orientation. Generally, DKI acquires images with multiple b-value shells but with less direction than Q-ball does, thus DKI provides favorable quantitative diffusion probability distribution, but Q-ball with a higher resolution angular distribution. Therefore, the advantage of the SD map is that it can be concomitantly obtained with a fiber crossing map using a single measurement of Q-ball, while angular resolution of AKC is generally too low to simultaneously produce a high quality fiber crossing map. Both SD and AKC are sensitive to early stages of white matter reorganization, and are superior to FA in the detection of white matter reorganization with prominent crossing axons [19], [37]. However, solving the ODF requires high angular resolution MRI data. The more directions the MRI scan contains, the more accurate the ODF produced, and the more time the MRI scan takes. Thus, very high angular resolution DKI and Q-ball DTI are rarely employed for in vivo studies.

SD and FA should show a similar pattern if the white matter bundle is well organized along a single direction [19]. The BLFB stained slices and MRI fiber crossing maps of the current study (Fig. 5) showed that the majority of axonal bundles were well organized into a single direction and are present only in small areas with crossing bundles. These results indicated that fiber crossing, at least 6 weeks after TBI, has a minor affect on FA values of TBI recovery cerebral tissue. Thus, the lower FA values in control rats than in the methamphetamine treated rats were primarily caused by fiber density rather than by fiber crossing, which was also alternatively confirmed by the significant Pearson’s correlation (R = 0.54, p<0.02) between the FA measured 6 weeks after TBI and histological BLFB measurements. The temporal profiles of FA measurements (Fig. 2 c), suggested that low dose methamphetamine treatment after TBI improves white matter reorganization from 5 weeks after brain traumatic insult in rat, compared with saline treatment.

Immunocytochemically, SMI 32 visualizes neuronal cell bodies, dendrites and some thick axons, especially for neocortical pyramidal neurons [53]. The significant increase of SMI-32 and MAP-2 immunoreactive proteins, thus, suggests that methamphetamine treatment after TBI promotes the development of dendrites. Meanwhile, the treatment also increased axonal myelination, which was indicated by a significant increase of BLFB immunoreactive axons in the ipsilateral hemisphere of TBI rats. Therefore, these results indicated that methamphetamine treatment improves neuronal recovery in TBI rats. However, the synaptophysin data did not show any significant differences between the treated and control rats, and the reason for this is unclear.

Neuronal fiber reorganization after injury may play an important role in fuctional recovery [41], [42]. We found that 12 hour post stroke methamphetamine treatment at an infusion dose of 1.0 mg/kg/hr for 24 hours in rats did not reduce infarct volume examined at 7 days post injury, but, did improve neurological scores in rats compared with saline treated controls [18]. In the present study, our data demonstrated that low dose methamphetamine treatment of TBI reduces neither TBI lesion volume, nor ventricular volume expansion, but, the treatment increased white matter reorgnization, and hence, functional outcomes over a 6 week period after the TBI insult, compared to the control. This result indicated that, as in the stroke model, delayed administration of very low dose methamphetamine has a minor effect on neuroprotection, but yet still improves functional outcome in TBI rats; which implies that reorganization of white matter may be a major contributor to the functional recovery after brain injury. The improved functional outcome with low-dose methamphetamine treatment also suggests that a significant increase in cortical neuron activity in methamphetamine treated rats may enhance sensorimotor functions, such as mNSS and foot-fault, without improving cognitive function, as measured with the Morris Water Maze test.

Using a different treatment protocol (0.845 mg/kg in bolus injection, followed by 0.5 mg/kg/hr×24 hrs infusion), timing (3 hrs after TBI) and traumatic model [15], methamphetamine treatment of TBI in rat was recently reported to improve not only behavioral function in mNSS and foot fault tests, but also cognitive function in the Morris Water Maze test 27 days after TBI insult. Thus, combined with our present results, these data suggest that the functional recovery after TBI in rats is dependent on dose and time of methamphetamine treatment.

Methamphetamine is a potent psychostimulant that alters the release and reuptake of dopamine, norepinephrine, and serotonin [18]. In a transient middle cerebral artery occlusion model in the rat, low dose of methamphetamine was found to mediate neuroprotection through activation of a dopamine-PI3K-AKT signaling pathway [18]. Similar results were found in a rat lateral fluid percussion injury TBI model [15]. In the current study, the mean TBI lesion volume in the treated group (25.51±12.14 mm^3^) was smaller than that in control group (32.49±9.45 mm^3^) although it did not reach statistical significance at 6 weeks after TBI. We note, that the dose used in the present study was one tenth of that used in the earlier studies, and therefore a higher dose may produce a more robust effect [15]. Therefore, the very low dose methamphetamine treatment of rat initiated at 8 hours post TBI may provide a minor neuroprotective effect. Several previous studies have demonstrated increased axonal density detected by MRI FA and corresponding BLFB staining [19], [23], [37]–[39], and cell-based treatment 6 hours after TBI demonstrated neurorestorative effects compared with untreated animals [54]. Thus, based on the MRI FA detected increase of axonal bundles and confirmed with corresponding BLFB staining data, the low dose methamphetamine treatment may promote neurorestorative effects, since it induces white matter reorganization and functional recovery after TBI. However, the exact mechanisms underlying the therapeutic effects of low-dose methamphetamine for TBI are not clear, and merit further investigation.

In summary, very low dose (a bolus injection of 0.42 mg/kg followed by an infusion of 0.05 mg/kg/hr for 24 hrs) methamphetamine treatment starting at 8 hours after TBI in rats did not significantly reduce lesion volume and brain atrophy, however, it did affect white matter structure and remodeling, and neurological function at 6 weeks after traumatic insult. In addition, MRI provided sensitive and insightful indices underlying this functional benefit.
